# Visualization of specific repetitive genomic sequences with fluorescent TALEs in *Arabidopsis thaliana*

**DOI:** 10.1093/jxb/erw371

**Published:** 2016-10-06

**Authors:** Satoru Fujimoto, Shigeo S. Sugano, Keiko Kuwata, Keishi Osakabe, Sachihiro Matsunaga

**Affiliations:** ^1^Department of Applied Biological Science, Faculty of Science and Technology, Tokyo University of Science, Noda, Chiba 278–8510, Japan; ^2^Graduate School of Science, Kyoto University, Kyoto 606–8502, Japan; ^3^PRESTO, JST, Saitama 332-0012, Japan; ^4^Institute of Transformative Bio-Molecules, Nagoya University, Nagoya 464–8601, Japan; ^5^Faculty of Bioscience and Bioindustry, Tokushima University, Tokushima 770–8513, Japan

**Keywords:** Centromere, chromatin, fluorescent protein, genome editing, live cell imaging, rDNA, telomere, transcription activator-like effector.

## Abstract

Live imaging of the dynamics of nuclear organization provides the opportunity to uncover the mechanisms responsible for four-dimensional genome architecture. Here, we describe the use of fluorescent protein (FP) fusions of transcription activator-like effectors (TALEs) to visualize endogenous genomic sequences in *Arabidopsis thaliana.* The ability to engineer sequence-specific TALEs permits the investigation of precise genomic sequences. We could detect TALE-FP signals associated with centromeric, telomeric, and rDNA repeats and the signal distribution was consistent with that observed by fluorescent *in situ* hybridization. TALE-FPs are advantageous because they permit the observation of intact tissues. We used our TALE-FP method to investigate the nuclei of several multicellular plant tissues including roots, hypocotyls, leaves, and flowers. Because TALE-FPs permit live-cell imaging, we successfully observed the temporal dynamics of centromeres and telomeres in plant organs. Fusing TALEs to multimeric FPs enhanced the signal intensity when observing telomeres. We found that the mobility of telomeres was different in sub-nuclear regions. Transgenic plants stably expressing TALE-FPs will provide new insights into chromatin organization and dynamics in multicellular organisms.

## Highlight

Transcription activator-like effectors fused to fluorescent proteins can visualize repetitive genomic sequences including centromere, telomere, and rDNA sequences for analysing chromatin dynamics in living plant cells.

## Introduction

Genome organization is a dynamic process that influences gene expression, genome maintenance, and responses to environmental stimuli in many organisms (Kurz *et al.*, 1996; Franklin and Cande, 1999; Cobbe and Heck, 2000; Misteli, 2007). Fluorescent *in situ* hybridization (FISH) is a powerful tool for determining the spatial organization of specific DNA sequences on chromosomes or in nuclei (Hans de Jong *et al.*, 1999). Although FISH is, in principle, capable of detecting any specific sequence, it cannot provide information about chromatin dynamics, because FISH requires the fixation of cells and a high-temperature treatment for hybridization. To visualize chromatin organization without fixation and hybridization, several *in vivo* imaging techniques have been developed. The first report describing *in vivo* imaging of chromatin used DNA-binding domains of bacterial transcription factors, such as the lac repressor (LacI) or tetracycline repressor (TetR) systems (Robinett *et al.*, 1996; Kato and Lam, 2001; Matzke *et al.*, 2005). After repeats of the cognate DNA sequences recognized by these transcription factors are introduced into specific genomic regions, the nuclear localization of these regions can be observed by expressing GFP-tagged transcription factors. Using this system, the relationship between chromatin conformation, gene expression (Rosin *et al.*, 2008) and DNA damage (Hirakawa *et al.*, 2015) have been examined in plants.

However, as the preceding method requires the random insertion of exogenous DNA into the genome, it is not amenable to *in vivo* imaging of specific genomic loci. In order to solve this problem, an *in vivo* imaging strategy that made use of zinc-finger domain containing proteins was developed (Lindhout *et al.*, 2007). These domains contain a DNA-binding motif whose specificity can be engineered and that have previously been used for genome editing. Engineered zinc-finger domains have been used to detect endogenous centromere sequences (Lindhout *et al*., 2007). However, because each modular zinc-finger protein recognizes a DNA triplet with context-dependent interactions between neighbouring zinc-fingers, engineering proteins to recognize specific sequences can be challenging (Gaj *et al.*, 2013). By contrast, TALEs contain a DNA binding domain comprising repeats of an approximately 34 amino acid-long DNA recognition module. Each module robustly recognizes a single nucleotide (Boch *et al.*, 2009; Moscou and Bogdanove, 2009). In human and mouse cultured cells, TALE-FPs (also known as TGV, TALEColor, and dTALE) have been successfully used for the visualization of chromatin (Ma *et al.*, 2013; Miyanari *et al.*, 2013; Thanisch *et al.*, 2014). Furthermore, transiently expressed TALE-FPs (TALE-light) have previously been used to analyse chromatin dynamics in *Drosophila melanogaster* (Yuan *et al.*, 2014). Here we present stable transgenic lines expressing TALE-FPs for centromeric, telomeric, and rDNA sequences and analyse the dynamics of these sequences in the nuclei of plant organs.

## Materials and methods

### Plasmid construction

TALEs were assembled in pZHY500 by Golden Gate assembly using the TAL effector toolbox (Cermak *et al.*, 2011) obtained from Addgene. The target sequences are provided in Supplementary Table S1 at *JXB* online. pCE1.2iFok was used for the construction of TALE-fluorescent protein (FP) fusion expression cassettes. pCE1.2iFok was obtained by cloning the expression cassette, the 2xCaMV35S promoter with the omega enhancer—the *Fok*I nuclease domain—and the *Arabidopsis HSP18.2* gene terminator into the pCR8/GW/TOPO TA Cloning vector (ThermoFisher Scientific). The assembled TALEs were cloned into pCE-N-GFP, pCE-N-Venus, pCE-N-tdTomato, or pCE-N-3xGFP. pCE-N-FPs were constructed by modifying pCE1.2iFok, including replacement of the promoter region with a nuclear localization signal sequence by *Asc*I and *Nco*I digestion and replacement of the *Fok*I domain with sGFP, Venus (Nagai *et al.*, 2002), tdTomato (Shaner *et al.*, 2004) or 3xGFP (Ma *et al.*, 2015) with an In-Fusion HD Cloning kit (Takara Bio Inc.). The sequence of pCE-N-GFP is provided in see Supplementary Fig. S1 at *JXB* online. The destination vector pGWB-RPS5a was obtained by modification of pGWB501 (Nakagawa *et al.*, 2007). The 1.7-kb *Sbf*I and *Xba*I fragment containing the RPS5a promoter (Weijers *et al.*, 2001) was inserted into the *Sbf*I–*Xba*I site of pGWB501 to yield pGWB-RPS5a. The TALE-FPs were transferred either to pGWB-RPS5a for microscopic observation or to pMDC32 (Curtis and Grossniklaus, 2003) for immunoprecipitation with LR clonase (Invitrogen). Details of vector construction are shown in Supplementary Fig. S2.

### Plant materials and transformation

The plasmids encoding engineered TALE-FP fusions were introduced into *Agrobacterium tumefaciens* GV3101. Transformation of either *A. thaliana* accession Col-0 or the KU70 mutant (SALK_123114) (Gámez-Arjona *et al.*, 2010) was performed by the floral dip method (Clough and Bent, 1998). The transformants were selected on solidified 1/2 MS medium containing claforan and hygromycin.

### Immunolabelling of TALE-GFP and FISH analysis

Indirect immunofluorescence was performed on root cells or isolated nuclei from leaves as described previously (Fujimoto *et al.*, 2004; Tirichine *et al.*, 2009). CENH3 was detected with rabbit anti-HTR12 antibody (Talbert *et al.*, 2002) (1:1 000) and GFP was detected with either rat anti-GFP antibody (1:200 Bio Academia 1A5) or rabbit anti-GFP antibody (1:200 Invitrogen A11122). The signals were amplified using Alexa 488 anti-rat antibody (1:200, Life Technologies) and Alexa 546 anti-rabbit antibody (1:200, Life Technologies) or Alexa 488 anti-rabbit antibody (1:200, Life Technologies). Cells were counterstained with DAPI in VECTASHIELD anti-fade mounting medium (Vector Laboratories).

FISH was performed essentially as described previously by Shibata and Murata (2004) following immunofluorescence staining. Briefly, probes recognizing 180bp centromeric repeats or telomeres were synthesized by nick translation using a DIG nick translation mix (Roche Diagnostics). The DIG-labelled probes were visualized using anti-digoxigenin-rhodamine Fab fragments (Roche).

### Microscopy

For immunostaining and FISH, we used an upright microscope (BX53, Olympus, Tokyo, Japan) with a ×100 objective (UPLSAPO 100×, Olympus, Tokyo, Japan) and a CCD camera (DOC CAM U3-50S5M-C, Molecular Devices) controlled with MetaVue (Molecular Devices). For live imaging, we used an inverted microscope (IX81, Olympus, Tokyo, Japan) equipped with a spinning-disk confocal system (CSU-X disk, Yokogawa Electric, Tokyo, Japan) with ×40, ×60 or ×100 objectives (UPLFLN 40XO, PLAPO 60XO2PH, PLAPO 100XO) and a CMOS camera (Neo 5.5 sCMOS, ANDOR) controlled with MetaMorph (Molecular Devices). GFP and tdTomato were excited with 488nm and 561nm lasers, respectively. T_2_ populations were sown on 1/2 MS medium in Petri dishes and grown for 5 d prior to analysis, with the exception of floral organ samples. Flower buds were used for floral organ samples. Whole plants or floral organs were placed between cover slips mounted with water. For time-lapse observation, T_2_ populations were sown on solidified 1/2 MS medium in glass-bottomed dishes and grown for 7–14 d prior to imaging with confocal microscopy. Images were acquired every 2–5min using multiple *z*-planes (0.6–1.0-μm intervals) and 10–20 planes per sample were collected. For higher-resolution imaging, we used a confocal microscope (FV1200, Olympus, Tokyo, Japan) with a ×60 or a ×100 objective (UPLSAPO 60XW, UPLSAPO 100XO) and GaAsP detectors controlled with FluoView (Olympus, Tokyo, Japan). GFP, Venus, and 3xGFP were excited with a 473nm laser and tdTomato was excited with a 559nm laser. Images were processed by Fiji to generate maximum intensity projection images, adjust the contrast, reduce the background, and to add colour.

### Tracking analysis

TALE_telo-FP signals were plotted and the centres of the nuclear masses were calculated from the projection images of the time points (30min). The mean-squared displacement (MSD) was calculated from the average change in distance between each TALE-FP signal and the nuclear centre of mass over the possible combinations of lag time (Δ*t*). MSD values of the signals were averaged and the standard deviations were presented.

### Co-immunoprecipitation and MS analysis

Seedlings of *A. thaliana* expressing TALE_180-GFP or TALE_telo C-GFP were used for co-immunoprecipitation. Two grams of 3-week-old seedlings were fixed with 1% formaldehyde for 30min, ground with a mortar and pestle, and lysed in immunoprecipitation buffer containing 50mM HEPES-KOH (pH 7.5), 140mM NaCl, 1mM EDTA, 1% Triton X-100, 0.1% sodium deoxycholate, 1mM PMSF, and Complete Protease Inhibitor Cocktail (Roche) (Mérai *et al.*, 2014). The homogenates were filtered through two layers of Miracloth to remove cell debris. The flow-through fraction was sonicated with Bioruptor UCD-250 for 15min at the middle intensity (200 W, 15/30s ON/ OFF cycle) to shear chromatin and debris was then removed by centrifugation at 15 000×*g* for 30min. The supernatant was used for immunoprecipitation with GFP trap magnetic beads. Washes were performed with immunoprecipitation buffer. SDS-PAGE was carried out according to the method described by Laemmli (1970). Co-immunoprecipitated samples were dissolved in sample buffer and resolved (~2cm) using a slab gel. Each lane was divided into six pieces. In-gel digestion was performed according to the method described by Rosenfeld *et al.*(1992).

Samples were analysed by nano-flow reverse phase liquid chromatography followed by tandem MS, using a Triple TOF 5600+ (AB SCIEX, Concord, Canada). A capillary reverse phase HPLC-MS/MS system composed of an Eksigent Ekspert nano-LC 400 HPLC system (AB SCIEX) connected directly to an AB SCIEX quadrupole time-of-flight (QqTOF) TripleTOF 5600+ mass spectrometer (AB SCIEX) in the trap and elute mode was used to identify the ensemble of proteins present in each sample. In the trap and elute mode, the samples were automatically injected using the Ekspert 400 system into a peptide trap column (ChromeXP, C18-CL, 200 µm I.D. ×0.5mm, 3 µm particle size, 120 Å pore size, AB SCIEX) attached to a cHiPLC system (AB SCIEX) for desalting and the concentration of peptides. After washing the trap with MS-grade water containing 0.1% trifluoroacetic acid and 2% acetonitrile (solvent C), the peptides were loaded into a separation capillary reverse phase column (ChromeXP, C18-CL, 75 µm I.D. ×150mm, 3 µm particle size, 120 Å pore size, AB SCIEX) by switching the valve. The eluents used were: (A) 100% water containing 0.1% formic acid and (B) 100% acetonitrile containing 0.1% formic acid. The column was developed at a flow rate of 0.5 μl min^–1^ with the following concentration gradient of acetonitrile: from 2% B to 32% B in 100min, 32% B to 80% B in 1min, sustaining 80% B for 10min, from 80% B to 2% B in 1min, and finally re-equilibrating with 2% B for 15min. Mass spectra and tandem mass spectra were recorded in positive-ion and ‘high-sensitivity’ mode with a resolution of ~35 000 full-width half-maximum. The nanospray needle voltage was typically 2 300 V in HPLC-MS mode. After the acquisition of approximately six samples, TOF MS spectra and TOF MS/MS spectra were automatically calibrated during dynamic LC-MS and MS/MS autocalibration acquisitions by injecting 50fmol BSA. The Analyst TF1.6 system (AB SCIEX) was used to record peptide spectra over the mass range of *m/z* 400–1250, and MS/MS spectra in information-dependent data acquisition over the mass range of *m/z* 100–1600. For CID-MS/MS, the mass window for precursor ion selection of the quadrupole mass analyser was set to 0.7±0.1Da. The precursor ions were fragmented in a collision cell using nitrogen as the collision gas. Advanced information-dependent acquisition (IDA) was used for MS/MS collection on the TripleTOF 5600+ to obtain MS/MS spectra for the 20 most abundant parent ions following each survey MS1 scan (250ms acquisition time per MS1 scan, and typically 100ms acquisition time per MS/MS). Dynamic exclusion features were based on *m/z* value and were set to an exclusion mass width of 50 mDa and an exclusion duration of 12s.

Searches were performed by using the Mascot server version 2.4.0 (Matrix Science, MA, USA) against the latest Swissprot database for protein identification. Search parameters were set as follows: the enzyme selected was used with three maximum missing cleavage sites, species limited to *Arabidopsis thaliana* (thale cress), a mass tolerance of 45ppm for peptide tolerance, 0.1Da for MS/MS tolerance, fixed modification of carbamidomethyl (C), and variable modification of oxidation (M). The maximum expectation value for accepting individual peptide ion scores [−10*Log(*P*)] was set to ≤0.05, where *P* is the probability that the observed match is a random event. Protein identification and modification information returned from Mascot were manually inspected and filtered to obtain confirmed protein identification and modification lists of CID MS/MS.

## Results and discussion

### Visualization of endogenous genomic sequences of *A. thaliana* with fluorescent TALEs

**Fig. 1.**
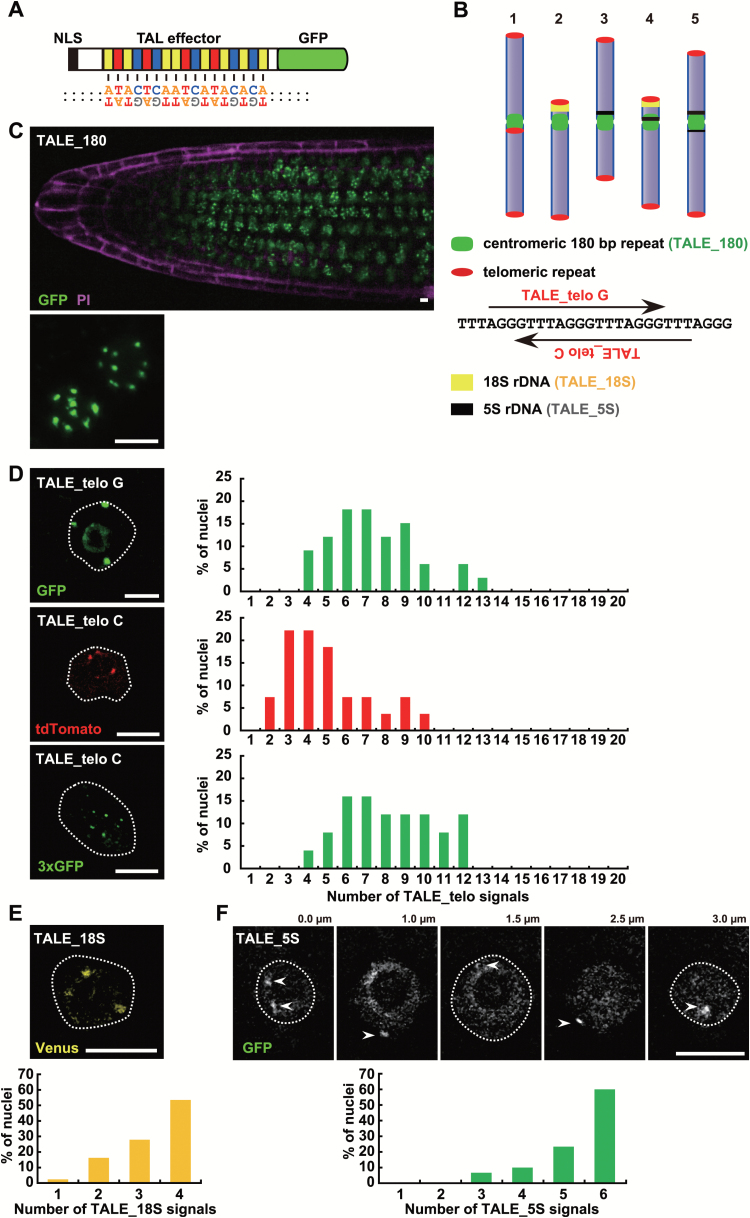
Visualization of endogenous genomic sequences with fluorescent TALEs in *A. thaliana*. (A) Schematic illustration of TALE_180-GFP for centromere visualization of *A. thaliana*. (B) Schematic illustration of the distribution of repetitive sequences on five chromosomes in *A. thaliana*. Two TALEs, recognizing the telomere G strand (TALE_telo_G) and C strand (TALE_telo_C), respectively, were used for telomere visualization. (C) A fluorescent image of the root of a transgenic line stably expressing TALE_180-GFP. The image is a projection image. The root was counterstained with propidium iodide which stains cell walls magenta (top). The left region exhibits a root tip. Two meristematic cells are shown in a magnified image (bottom). (D) Fluorescent images of nuclei expressing TALE_telo_G-GFP (top), TALE_telo_C-tdTomato (middle), and TALE_telo_C-3× GFP (bottom). The distribution of TALE_telo signals in a nucleus is shown in the graph (right). *n*=33 (TALE_telo G-GFP), 27 (TALE_telo C-tdTomato) and 25 (TALE_telo C-3×GFP). (E) Fluorescent images of nuclei expressing TALE recognizing 18S rDNA with Venus (top). The distribution of TALE_18S-Venus signals in a nucleus is shown in the graph (bottom). *n*=43. (F) Fluorescent images of nuclei expressing TALE recognizing 5S rDNA with GFP. Arrowheads indicate 5S rDNA signals (top). The distribution of TALE_5S-GFP signals in a nucleus (bottom). *n*=30. White dot lines indicate nuclear membranes. Scale bars, 5 μm.

To image endogenous genomic sequences in plant nuclei, we designed a TALE protein fused with a FP (TALE-FP) (Fig. 1A). We first investigated the localization of centromeres by targeting the 180bp centromeric repeats known as pAL1 (Fig. 1B). This sequence belongs to the major centromeric satellite, forms tandem arrays approximately 2 Mbp in length, and occupies the centromeric regions of all chromosomes in *A. thaliana* (Copenhaver *et al.*, 1999; Haupt *et al.*, 2001). Such repetitive sequences permit the recruitment of multiple molecules of TALE-FP to the same locus. To construct TALEs that recognize 17 nt of the 180bp centromeric repeat sequence (TALE_180) (Supplementary Table S1), we cloned DNA encoding these TALEs into a vector with an in-frame fusion to a FP under the RPS5a promoter. *A. thaliana* was transformed with these constructs using the floral dip method. Figure 1C (top) shows the root of a transformant stably expressing TALE_180-GFP. Ten discrete signals were usually detected in each nucleus of meristematic root cells, each presumably corresponding to one of the 10 centromeres of *A. thaliana* chromosomes (Fig. 1C).

To address whether the TALE-FP system can be used to visualize other repetitive genomic sequences, we next designed TALEs targeting the C-strand or G-strand of the telomeres, the 18S rDNA, or the 5S rDNA repeats (Fig. 1D–F; Supplementary Table S1) and fused them to FPs (TALE_telo C-tdTomato, TALE_telo G-GFP, TALE_18S-Venus, and TALE_5S-GFP, respectively). We detected small, dot-like signals presumably corresponding to telomeres in cells expressing either of the TALE_telo-FP fusions (Fig. 1D). In addition to small dot-like signals, two strong signals were observed in cells expressing TALE_telo G-GFP. We constructed a transgenic line expressing TALE_telo C-GFP, where we detected only weak signals of TALE_telo C-GFP. Thus, we constructed TALE_telo C-tdTomato, which is a red fluorescent protein that forms tandem dimers, and could detect TALE_telo C-tdTomato signals (Fig. 1D). However, the signal intensity of TALE_telo C-tdTomato was less than that observed in lines expressing TALE_telo G-GFP, despite repeated rounds of selection to isolate transgenic lines with strong expression of the TALE_telo C-tdTomato.

Therefore, to enhance the signal, we constructed a 3×GFP fusion for TALE_telo C visualization. TALE_telo C-3×GFP exhibited signal intensities comparable with TALE_telo G-GFP (Fig. 1D). This improvement demonstrated that the use of tandemly repeated FPs, at least up to trimers, is useful to enhance TALE-FP signals. All TALE_telo-FPs showed signal numbers lower than the expected maximum number (20 signals) per nucleus. Because FISH, using probes directed against telomeric repeats, also shows a similar distribution of signals (Schubert *et al.*, 2012), it is possible that close physical association of telomeres in *A. thaliana* (Feng *et al.*, 2014) reduces the number of discrete signals that were observed, rather than a shortcoming in our method.

We also used fluorescent TALE fusions to observe rDNA repeats. When we visualized the 18S rDNA with TALE_18S-Venus, one to four bright signals were observed around the nucleoli (Fig. 1E). In the case of TALE_5S-GFP-mediated visualization, we observed three to six weak signals in the nuclei (Fig. 1F). This distribution of signal intensity of TALE-FP fusions specific for rDNA is consistent with the fact that 18S rDNA genes are located on chromosomes 2 and 4 (Murata *et al.*, 1997; Fransz *et al.*, 2002) while 5S rDNA genes are near the centromeres of chromosomes 3, 4, and 5 (Murata *et al.*, 1997; Cloix *et al.*, 2000).

Some of our transgenic lines exhibited background fluorescence in the nucleoli or throughout the cytoplasm which may have been derived from unbound or off-target TALE-FPs. To combat this, we performed repeated selection to recover lines with an improved signal-to-noise ratio. Transgenic lines with highly expressed and specifically localized TALE_180-GFP were readily recovered but lines expressing TALE_rDNAs with low background were more difficult to acquire.

### Confirmation of plant TALE-FP localization by immunofluorescence and FISH

**Fig. 2.**
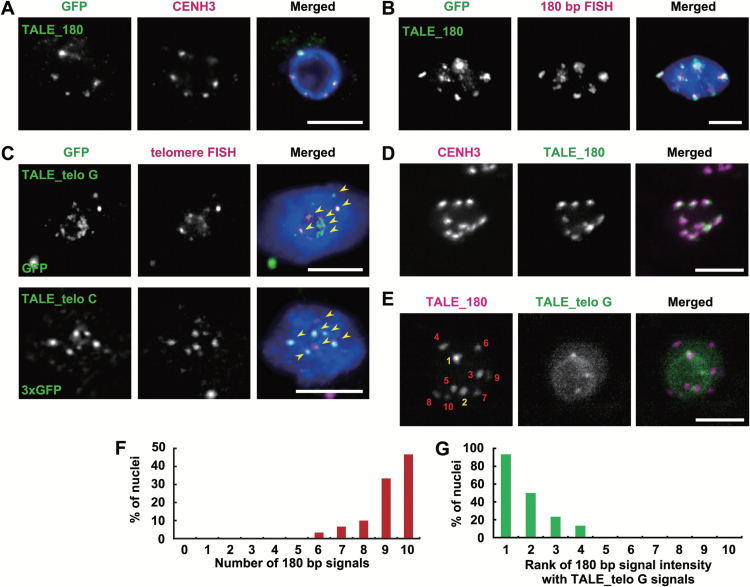
Confirmation of TALE-FP localization by immunostaining and FISH. (A) Fluorescent images of TALE_180-GFP and CENH3 immunostaining in a nucleus of an *A. thaliana* root cell expressing TALE_180-GFP. (B) Fluorescent images of GFP immunostaining signals and FISH signals targeting 180bp centromeric repeats in a nucleus of *A. thaliana* expressing TALE_180-GFP. (C) Fluorescent images of GFP immunostaining signals and FISH signals recognizing telomere repeats in a nucleus of *A. thaliana* expressing TALE_ telo G-GFP (top) or telo C-3×GFP (bottom). Arrowheads indicate the co-localization of the GFP and telomere signals. (D) Live cell imaging for TALEs recognizing 180bp centromeric repeats (TALE_180-GFP) and CENH3 (tdTomato). (E) The fluorescent image of a nucleus of living *A. thaliana* expressing both TALE_180-tdTomato and TALE_telo G-GFP. Numbers in the 180bp panel denote the rank of the signal intensity. Yellow and red numbers indicate TALE_180-tdTomato signals co-localized with or without TALE_telo G-GFP, respectively. Scale bars in (A)–(E), 5 μm. (F) Distribution of the number of TALE_180-tdTomato signals in a nucleus. *n*=30. (G) Rank of the signal intensity of TALE_180-tdTomato co-localized with telomere signals. Rank 1 is the strongest signal out of 10 TALE_180-tdTomato signals. *n*=30.

To verify the specific recognition of centromeres and telomeres by TALE-FP, we performed immunofluorescence and FISH to detect the repeat sequences. We analysed the centromere distribution by immunofluorescence using an anti-CENH3 antibody (Fig. 2A) or FISH using a 180bp repeat sequence as a probe (Fig. 2B). The centromere signals were widely dispersed and peripherally located in nuclei as reported by FISH (Armstrong *et al.*, 2001). The signals were co-localized with the signals of TALE_180-GFP. The distribution of telomeres was analysed by FISH using the telomere repeat sequence as a probe. FISH experiments previously reported that telomere signals of *A. thaliana* were mainly clustered around the nucleolus (Armstrong *et al.*, 2001). TALE_telo G and TALE_telo C signals were also distributed around the nucleolus and merged images showed that TALE_telo-FPs co-localized with the telomeres (Fig. 2C). Taken together, these results suggest that TALE-FPs are capable of performing reliable imaging of endogenous repetitive sequences in plants.

TALE-FPs also permit multiple colour imaging of genomic sequences. For imaging of centromeric DNA and a centromeric protein at the same time, we observed *A. thaliana* roots simultaneously expressing CENH3-tdTomato and TALE_180-GFP. In *A. thaliana*, CENH3 is a centromere-specific histone H3 variant that co-localizes with the 180bp repetitive sequences of all centromeres (Talbert *et al.*, 2002). Even though TALE_180-GFP co-localizes with CENH3, the signal intensity of TALE_180-GFP is not always consistent with that of CENH3 (Fig. 2D). This difference is presumably because TALE_180-GFP binds to both the core centromeric region as well as the pericentromeric region. Most CENH3 is thought to bind to 180bp repeats (Nagaki *et al.*, 2003); however, some clusters of 180bp repeats, particularly those that are long, are not fully occupied by CENH3 (Shibata and Murata, 2004). Previously, the lengths of the 180bp tandem repeats on each chromosome were estimated to be 2.26 Mbp (chromosome 1) to 1.4 Mbp (chromosome 3) from FISH experiments (Haupt *et al.*, 2001).

Next, we observed cells simultaneously expressing TALE_180-tdTomato and TALE_telo G-GFP. The strong signals associated with TALE_telo G-GFP were partially co-localized with TALE_180-tdTomato (Fig. 2E). The intensity of signals of TALE_180-tdTomato was compared among the nuclei of root meristematic cells. The 180bp signal intensity observed in centromeres associated with TALE_telo G is relatively high compared with those in other centromeres (Fig. 2F, G). Centromeric regions often contain long interstitial telomere sequences (ITSs) which consist of imperfect telomere-like repeats. *A. thaliana* has 349kb ITSs within the centromere of chromosome 1 (Richards *et al.*, 1991; Armstrong *et al.*, 2001; Vannier *et al.*, 2009). Telomere-associated proteins are specific for telomeres versus ITSs, suggesting that ITSs have no functional similarity with telomeres (Song *et al.*, 2008; Surovtseva *et al.*, 2009). The TALE_telo localizes not only to telomeres but also ITSs as TALE_telo associates with 2.6 telomeric repeats (Supplementary Table S1). Thus, we conclude that the bright spots observed in cells expressing TALE_telo G are the ITSs on chromosome 1. Moreover, the signals associated with the180bp repeats of chromosome 1 are larger in area than those for other chromosomes which is consistent with previous studies of the 180bp centromeric repeats by FISH (Haupt *et al.*, 2001). TALE_telo C and TALE_telo G are targeted to CCCTAAA and TTTAGGG repeats, respectively. Not only CG but also CHG and CHH are methylated in plant genomes. Furthermore, plant heterochromatin is enriched in methylated cytosines, in particular, methylated CHH (Vaquero-Sedas and Vega-Palas, 2014; Widman *et al.*, 2009). RVD HD shows a preference for cytosines, but not for methylated cytosines (Valton *et al.*, 2012). Therefore, targeting to the interstitial telomere sequences of TALE_telo C seems to be diminished compared with that of TALE_telo G. As a result of the application of different TALE repeats, the pattern of fluorescent signals was different between TALE_telo C and TALE_telo G.

### Identification of TALE-GFP binding histones by IP-MS

Recently, engineered DNA-binding molecule-mediated chromatin immunoprecipitation (enChIP) was developed for the purification of specific genomic regions using TALE (Fujita *et al.*, 2013). To confirm that our TALE-GFP fusions specifically interact with centromeres or telomeres, a GFP-trap was used to immunoprecipitate TALE-GFP interacting histones from whole extracts prepared from seedlings. As TALE_telo G exhibited association with ITSs, we used only TALE_telo C for the identification of telomere-associated histones. Mass spectrometry confirmed the presence of various histones (Table 1). Compared with the population of histones associated with TALE_telo C, TALE_180-associated histones were enriched for CENH3. Furthermore, histone H1 enrichment is less for TALE_telo C than for TALE_180. These data suggest that TALE_180 localizes to centromeres. Consistent with this, previous studies demonstrate that histone H1 binds to the compacted nucleosomal arrays of telomeres but with reduced stoichiometry relative to other genomic regions in *A. thaliana* (Ascenzi and Gantt, 1999).

**Table 1.** Histones co-immunoprecipitated with TALE–FPs

Histone	TALE_180		TALE_telo C	
	Peptide hits	Unique peptides	Peptide hits	Unique peptides
H1	34	6	3	1
H2A	61	17	27	10
H2B	83	7	20	4
H3	38	5	26	4
H4	65	8	35	5
CENH3	3	3	0	0

### Live observation of centromere and telomere dynamics *in vivo*

**Fig. 3.**
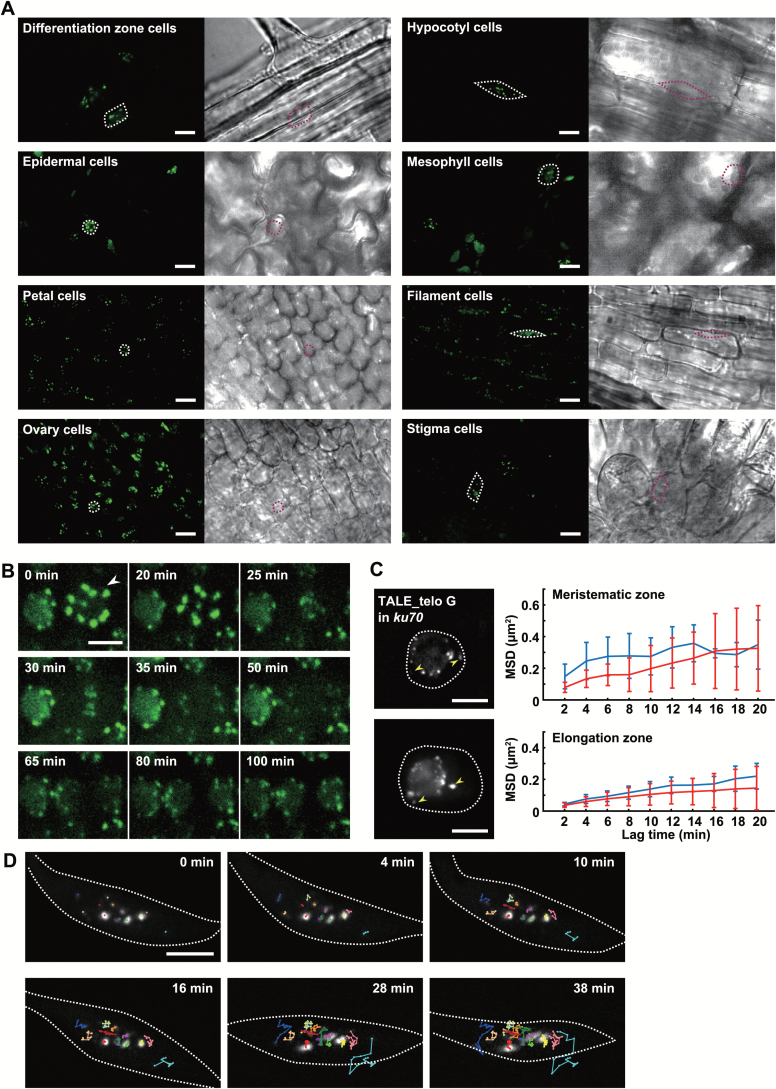
Live cell imaging of TALE-FPs in living tissues of *A. thaliana*. (A) Fluorescent images of TALE_180-GFP in nuclei of epidermal cells in the differentiation zone of a root, epidermal cells of a hypocotyl, epidermal cells on the abaxial side of a cotyledon, mesophyll cells of a cotyledon, epidermal cells of a petal, epidermal cells of a filament, epidermal cells of an ovary, and epidermal cells of a stigma. Each right-hand panel shows a phase contrast image. Scale bars, 10 μm. (B) Dynamics of TALE_180-GFP through mitosis in cells of the meristematic region of roots. The fluorescent images are constructed by maximum intensity projection. Arrowheads indicate a mitotic cell. (C) Fluorescent images of TALE_telo G-GFP in cells in meristematic (upper) and elongation (lower) zones of the root expressing TALE_telo G-GFP in *ku70* (left). Based on tracking analyses of TALE_telo G-GFP signals, the average of the MSD of each signal from single cells is shown with the standard deviation (right). The red and blue lines indicate signals associated with and without the nucleolus, respectively. White dotted lines indicate nuclear membranes. Yellow arrowheads indicate signals detached from the nucleolus. (D) Telomere dynamics in a root hair cell expressing TALE_telo C-tdTomato in *ku70*. The white dotted lines indicate nuclear membranes. Tracking lines of each TALE_telo C signal are shown by different colours. The images are constructed by maximum intensity projection. Scale bars in (B)–(D), 5 μm.

One of the advantages of plant TALE-FP is that it permits the analysis of chromatin organization in nuclei without disrupting the cell wall or permeabilizing the cell membrane during fixation like FISH. To ascertain whether plant TALE-FP is effective in various organs, we observed the signals of TALE_180-GFP in nuclei of cells in various organs. Clear and distinct fluorescent signals derived from TALE_180-GFP were observed in the nuclei of various organs including roots, hypocotyls, leaves, and floral organs (Fig. 3A). The fluorescence intensity of TALE-FP varies across plant organs unlike that observed in animal cultured cells because the nuclear size and morphology can be dynamically changed by endoreduplication and vacuole expansion (Matsunaga *et al.*, 2013). In addition, the intensity of TALE-FP signals is influenced by light scattering and absorption dependent on the imaging depth, unlike the flat cultured cells that adhere to dishes.

Plant TALE-FP also allows tracking of native chromatin mobility in living cells without inserting artificial reporter DNA sequences in the genome. We next analysed the chromatin dynamics throughout the cell cycle by time-lapse imaging of cells expressing TALE_180-GFP. TALE_180-GFP accumulates at centromeric regions throughout mitosis, albeit at diminished levels from prometaphase to telophase (Fig. 3B;see Supplementary Movie S1 at *JXB* online). The diminishment on mitotic chromosomes suggests that chromatin compaction may prevent the binding of TALE-GFP to its target sequences. As mentioned above, telomeric repeats could be detected using TALE-FP fusions that specifically recognize telomeres but the signal intensity was insufficient for time-lapse imaging. *Ku70* mutants of *A. thaliana* deregulate the control of telomere length resulting in elongation of telomeres to 20 kbp (Bundock *et al.*, 2002; Riha *et al.*, 2002). When TALE_telo FPs were expressed in *ku70* plants, the signals were stronger than in wild-type plants (Fig. 3C; Supplementary Fig. S3). The signals were observed as distinct puncta around the nucleolar periphery with low observable background signals in the nucleoplasm (Fig. 3C; Supplementary Fig. S3). This suggests that the elongation of telomeres increases the binding of TALE_telo FPs with telomeres which increases the signal-to-noise ratio. Using the *ku70* background, the mean-squared displacement (MSD) of telomeres was determined in meristematic cells and cells of the elongation zone of roots. Time-lapse observation of TALE_telo G-GFP in root cells shows that the movement of telomere signals differed slightly between nuclei. The movement of telomere signals was dependent on association with the nucleolus (Fig. 3C; Supplementary Fig. S3; Supplementary Movie S2). Observation of TALE_telo C-tdTomato in a nucleus of a root hair cell demonstrates that the mobility of each telomere was different. In addition, a few telomeres temporarily formed a cluster (Fig. 3D; Supplementary Movie S3), demonstrating the telomere association described in Fig. 1D. To our knowledge, this is the first report of telomere dynamics in living plants.

Here, we report for the first time the successful construction of stable transgenic lines to visualize endogenous genomic loci in a living multicellular organism using TALE-FPs. These stable lines do not need the injection of TALE-FPs and retain the signal intensity even after long time-lapse observation following repeated cell divisions during organ development and differentiation. This method overcomes the limitations of previous chromatin analyses by FISH, such as the fixation of nuclei, hybridization with DNA probes, and permeabilization of cell walls. The observation of plant TALE-FPs without the isolation or squashing of nuclei enables us to analyse chromatin dynamics in three- or four-dimensions in several organs while retaining plant morphology. The convenient distribution of seeds of stably transgenic lines will contribute to progress in the analyses of chromatin dynamics in the nuclei of multicellular organs and tissues. Therefore, plant transgenic lines expressing TALE-FPs will be a powerful tool to investigate spatiotemporal chromatin organization during various events including development, fertilization, environmental responses, and microbe interaction. Further refinement of genome visualization with genome editing techniques in plants will permit the monitoring of single loci, mutation sites, and nucleotide polymorphisms which is essential for epigenetic analyses and molecular breeding.

## Supplementary data

Supplementary data can be found at *JXB* online.

Figure S1. Nucleotide sequence of pCE-N-GFP.

Figure S2. Construction of TALE-FP expression vector.

Figure S3. Maximum intensity projections of TALE_telo G-GFP.

Table S1. Target sequences of TALE-FP.

Movie S1. Time-lapse observation of 180bp repeats through mitosis every 5min in root meristematic cells.

Movie S2. Time-lapse observation of telomere repeats in a root elongation zone cell.

Movie S3. Time-lapse observation of telomere repeats in a root hair cell.

Supplementary Data
