# Supplementary material for: Visualization of specific repetitive genomic sequences with fluorescent TALEs in Arabidopsis thaliana
Source: J Exp Bot. 2016 Oct 6;67(21):6101–10. doi: 10.1093/jxb/erw371 (PMC5100022; doi:10.1093/jxb/erw371)
Supplement: Supplementary Data [file supp_67_21_6101__index.html]

Visualization of specific repetitive genomic sequences with fluorescent TALEs in Arabidopsis thaliana — Visualization of specific repetitive genomic sequences with fluorescent TALEs in Arabidopsis thaliana — Supplementary Data 

# Visualization of specific repetitive genomic sequences with fluorescent TALEs in *Arabidopsis thaliana*

## Supplementary Data

Data files

- supplementary\_table\_S1\_figures\_S1\_S3.pdf - Supplementary Data
- supplementary\_Movie\_S1.avi - Supplementary Data
- supplementary\_Movie\_S2.avi - Supplementary Data
- supplementary\_Movie\_S3.avi - Supplementary Data
