# Supplementary material for: Visualization of specific repetitive genomic sequences with fluorescent TALEs in Arabidopsis thaliana
Source: J Exp Bot. 2016 Oct 6;67(21):6101–10. doi: 10.1093/jxb/erw371 (PMC5100022; doi:10.1093/jxb/erw371)
Supplement: Supplementary Data [file supp_erw371_supplementary_table_S1_figures_S1_S3.pdf]

Fujimoto et al.

Supplementary data

Movie S1 Time-lapse observation of 180 bp repeats through mitosis. Time-lapse observation of 180 bp repeats through mitosis every 5 min in root meristematic cells. Maximum intensity projection images of TALE_180-GFP signals of mitotic cells were assembled into the movie.

Movie S2 Time-lapse observation of telomere repeats in root elongation zone cell. Maximum intensity projection images of cells shown in Fig. 3C were assembled into the movie.

Movie S3 Time-lapse observation of telomere repeats in root hair cell. Maximum intensity projection images of cells shown in Fig. 3D cell were assembled into the movie (first round). Each signal was tracked (second round).

Table S1 Target sequences of TALE-FP.

	Target sequence
**TALE_180**	ATACTCAATCATACACA
**TALE_telo C**	AAACCCTAAACCCTAAAC
**TALE_telo G**	AGGGTTTAGGGTTTAGGG
**TALE_18S**	AGAGCTAATACGTGCAA
**TALE_5S**	GCGATCATACCAGCACT

```
   1 AAAAGGCCATCCGTCAGGATGGCCTTCTGCTTAGTTTGATGCCTGGCAGTTTATGGCGGGCGTCCTGCCCGCCACCCTCCGGGCCGTTGC
  91 TTCACAACGTTCAAATCCGCTCCCGGCGGATTTGTCCTACTCAGGAGAGCGTTCACCGACAAACAACAGATAAAACGAAAGGCCCAGTCT
 181 TCCGACTGAGCCTTTCGTTTTATTTGATGCCTGGCAGTTCCCTACTCTCGCGTTAACGCTTGCATGGATGTTTTCCCAGTCACGACGTTG
 271 TAAAACGACGGCCAGTCTTAAGCTCGGGCCCCAAATAATGATTTTATTTTGACTGATAGTGACCTGTTCGTTGCAACAAATTGATGAGCA  attL1
 361 ATGCTTTTTTATAATGCCAACTTTGTACAAAAAAGCAGGCTGGCGCGATGGCTCCTAAGAAAAAGCGCAAAGTCGGTATCCATGGCGTTC  NLS
 451 CCTCTAGATAACGCAGGATCCATGGTGAGCAAGGGCGAGGAGCTGTTCACCGGGGTGGTGCCCATCCTGGTCGAGCTGGACGGCGACGTA
         XbaI       BamHI
 541 AACGGCCACAAGTTCAGCGTGTCCGGCGAGGGCGAGGGCGATGCCACCTACGGCAAGCTGACCCTGAAGTTCATCTGCACCACCGGCAAG
 631 CTGCCCGTGCCCTGGCCCACCCTCGTGACCACCTTCACCTACGGCGTGCAGTGCTTCAGCCGCTACCCCGACCACATGAAGCAGCACGAC
 721 TTCTTCAAGTCCGCCATGCCCGAAGGCTACGTCCAGGAGCGCACCATCTTCTTCAAGGACGACGGCAACTACAAGACCCGCGCCGAGGTG
                                                                                                GFP
 811 AAGTTCGAGGGCGACACCCTGGTGAACCGCATCGAGCTGAAGGGCATCGACTTCAAGGAGGACGGCAACATCCTGGGGCACAAGCTGGAG
 901 TACAACTACAACAGCCACAACGTCTATATCATGGCCGACAAGCAGAAGAACGGCATCAAGGTGAACTTCAAGATCCGCCACAACATCGAG
 991 GACGGCAGCGTGCAGCTCGCCGACCACTACCAGCAGAACACCCCCATCGGCGACGGCCCCGTGCTGCTGCCCGACAACCACTACCTGAGC
1081 ACCCAGTCCGCCCTGAGCAAAGACCCCAACGAGAAGCGCGATCACATGGTCCTGCTGGAGTTCGTGACCGCCGCCGGGATCACTCACGGC
1171 ATGGACGAGCTGTACAAGTAATTCTTAATTAACCCAATTCTTAATTAACCCAATTCGACCCAGCTTTCTTGTACAAAGTTGGCATTATAA
                                                                                                attL2
1261 GAAAGCATTGCTTATCAATTTGTTGCAACGAACAGGTCACTATCAGTCAAAATAAAATCATTATTTGCCATCCAGCTGATATCCCCTATA
1351 GTGAGTCGTATTACATGGTCATAGCTGTTTCCTGGCAGCTCTGGCCCGTGTCTCAAAATCTCTGATGTTACATTGCACAAGATAAAAATA
1441 TATCATCATGCCTCCTCTGGACCAGCCAGGACAGAAATGCCTCGACTTCGCTGCTACCCAAGGTTGCCGGGTGACGCACACCGTGGAAAC
1531 GGATGAAGGCACGAACCCAGTGGACATAAGCCTGTTCGGTTCGTAAGCTGTAATGCAAGTAGCGTATGCGCTCACGCAACTGGTCCAGAA
1621 CCTTGACCGAACGCAGCGGTGGTAACGGCGCAGTGGCGGTTTTCATGGCTTGTTATGACTGTTTTTTTGGGGTACAGTCTATGCCTCGGG
1711 CATCCAAGCAGCAAGCGCGTTACGCCGTGGGTCGATGTTTGATGTTATGGAGCAGCAACGATGTTACGCAGCAGGGCAGTCGCCCTAAAA
1801 CAAAGTTAAACATCATGAGGGAAGCGGTGATCGCCGAAGTATCGACTCAACTATCAGAGGTAGTTGGCGTCATCGAGCGCCATCTCGAAC
1891 CGACGTTGCTGGCCGTACATTTGTACGGCTCCGCAGTGGATGGCGGCCTGAAGCCACACAGTGATATTGATTTGCTGGTTACGGTGACCG
1981 TAAGGCTTGATGAAACAACGCGGCGAGCTTTGATCAACGACCTTTTGGAAACTTCGGCTTCCCCTGGAGAGAGCGAGATTCTCCGCGCTG
                                                                                                SpeR
2071 TAGAAGTCACCATTGTTGTGCACGACGACATCATTCCGTGGCGTTATCCAGCTAAGCGCGAACTGCAATTTGGAGAATGGCAGCGCAATG
2161 ACATTCTTGCAGGTATCTTCGAGCCAGCCACGATCGACATTGATCTGGCTATCTTGCTGACAAAAGCAAGAGAACATAGCGTTGCCTTGG
2251 TAGGTCCAGCGGCGGAGGAACTCTTTGATCCGGTTCCTGAACAGGATCTATTTGAGGCGCTAAATGAAACCTTAACGCTATGGAACTCGC
2341 CGCCCGACTGGGCTGGCGATGAGCGAAATGTAGTGCTTACGTTGTCCCGCATTTGGTACAGCGCAGTAACCGGCAAAATCGCGCCGAAGG
2431 ATGTCGCTGCCGACTGGGCAATGGAGCGCCTGCCGGCCCAGTATCAGCCCGTCATACTTGAAGCTAGACAGGCTTATCTTGGACAAGAAG
2521 AAGATCGCTTGGCCTCGCGCGCAGATCAGTTGGAAGAATTTGTCCACTACGTGAAAGGCGAGATCACCAAGGTAGTCGGCAAATAACCCT
2611 CGAGCCACCCATGACCAAAATCCCTTAACGTGAGTTACGCGTCGTTCCACTGAGCGTCAGACCCCGTAGAAAAGATCAAAGGATCTTCTT
2701 GAGATCCTTTTTTTCTGCGCGTAATCTGCTGCTTGCAAACAAAAAAACCACCGCTACCAGCGGTGGTTTGTTTGCCGGATCAAGAGCTAC
2791 CAACTCTTTTTCCGAAGGTAACTGGCTTCAGCAGAGCGCAGATACCAAATACTGTCCTTCTAGTGTAGCCGTAGTTAGGCCACCACTTCA
2881 AGAACTCTGTAGCACCGCCTACATACCTCGCTCTGCTAATCCTGTTACCAGTGGCTGCTGCCAGTGGCGATAAGTCGTGTCTTACCGGGT
2971 TGGACTCAAGACGATAGTTACCGGATAAGGCGCAGCGGTCGGGCTGAACGGGGGGTTCGTGCACACAGCCCAGCTTGGAGCGAACGACCT  pUC ori
3061 ACACCGAACTGAGATACCTACAGCGTGAGCTATGAGAAAGCGCCACGCTTCCCGAAGGGAGAAAGGCGGACAGGTATCCGGTAAGCGGCA
3151 GGGTCGGAACAGGAGAGCGCACGAGGGAGCTTCCAGGGGGAAACGCCTGGTATCTTTATAGTCCTGTCGGGTTTCGCCACCTCTGACTTG
3241 AGCGTCGATTTTTGTGATGCTCGTCAGGGGGGCGGAGCCTATGGAAAAACGCCAGCAACGCGGCCTTTTTACGGTTCCTGGCCTTTTGCT
3331 GGCCTTTTGCTCACATGTTCTTTCCTGCGTTATCCCCTGATTCTGTGGATAACCGTATTACCGCCTTTGAGTGAGCTGATACCGCTCGCC
3421 GCAGCCGAACGACCGAGCGCAGCGAGTCAGTGAGCGAGGAAGCGGAAGAGCGCCCAATACGCAAACCGCCTCTCCCCGCGCGTTGGCCGA
3511 TTCATTAATGCAGCTGGCACGACAGGTTTCCCGACTGGAAAGCGGGCAGTGAGCGCAACGCAATTAATACGCGTACCGCGAGCCAGGAAG
3601 AGTTTGTAGAAACGCA
```

Figure S1 Nucleotide sequence of pCE-N-GFP. For the pCE-N-Venus, pCE-N-tdTomato and pCE-N-3xGFP, GFP gene was replaced with Venus, tdTomato, or 3xGFP.

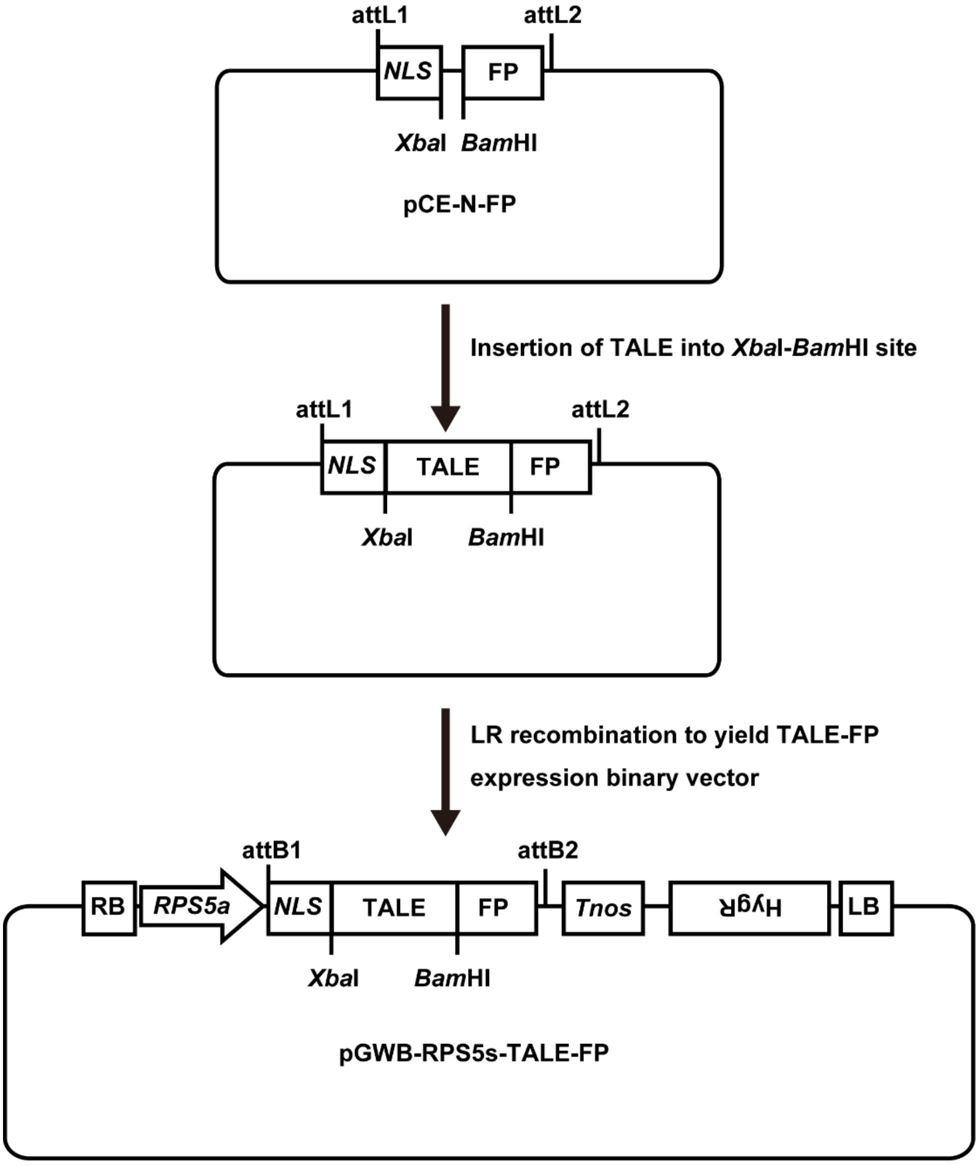

Figure S2 Construction of TALE-FP expression vector.

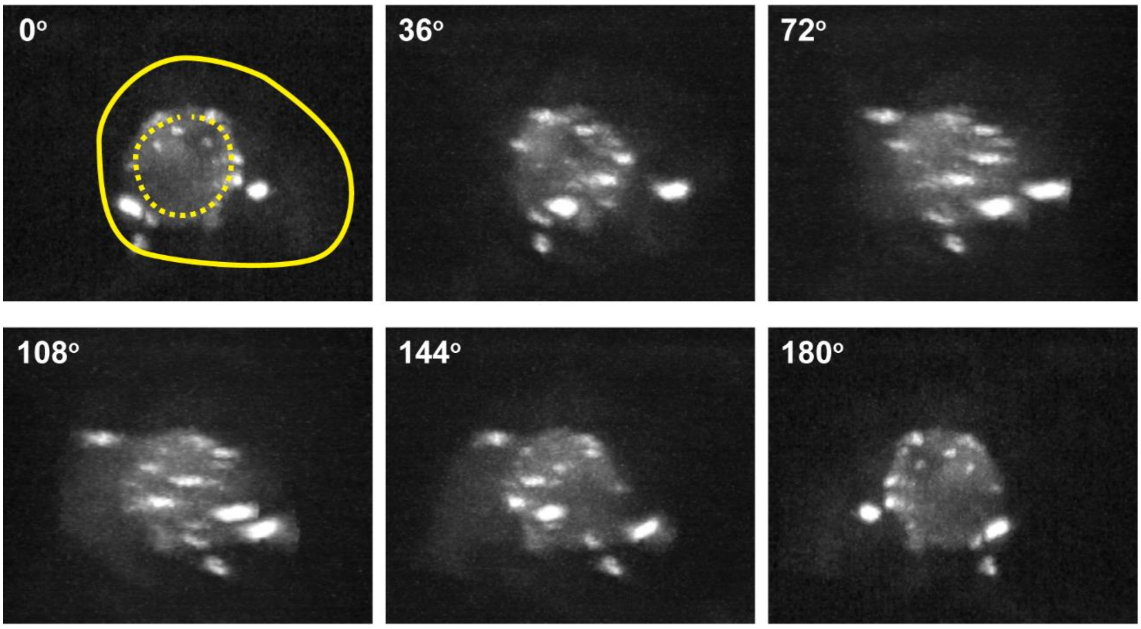

Figure S3 Maximum intensity projections of TALE_telo G-GFP. Projection images of TALE_telo G-GFP signals from root elongation zone cell from *ku70* plants (Fig. 3C) reconstructed from image sections with a z interval of 0.2 μm. Solid and dotted yellow lines indicate the edge of nucleus and nucleolus, respectively.
